# The Goldfish SG2NA Gene Encodes Two α-Type Regulatory Subunits for PP-2A and Displays Distinct Developmental Expression Pattern

**DOI:** 10.4137/grsb.s2764

**Published:** 2009-07-21

**Authors:** Hai-Li Ma, Yun-Lei Peng, Lili Gong, Wen-Bin Liu, Shuming Sun, Jiao Liu, Chun-Bing Zheng, Hu Fu, Dan Yuan, Junqiong Zhao, Pei-Chao Chen, Si-si Xie, Xiao-Ming Zeng, Ya-Mei Xiao, Yun Liu, David Wan-Cheng Li

**Affiliations:** 1 Key Laboratory of Protein Chemistry and Developmental Biology of Education Ministry of China, College of Life Sciences, Hunan Normal University, Changsha, Hunan, 410081, China; 2 Departments of Biochemistry and Molecular Biology and; 3 Ophthalmology and Visual Sciences, College of Medicine, University of Nebraska Medical Center, Omaha, NE 68198, USA

**Keywords:** goldfish, PP-2A, SG2NA, gene expression, molecular cloning, development, dephosphorylation, retina, lens

## Abstract

SG2NA is a member of the striatin protein family. In human and mouse, the SG2NA gene encodes two major protein isoforms: SG2NAα and SG2NAβ. The functions of these proteins, except for acting as the regulatory subunits for PP-2A, remain largely unknown. To explore the possible functions of SG2NA in lower vertebrates, we have isolated two SG2NA cDNAs from goldfish, *Carassius auratus.* Our results reveal that the first cDNA contains an ORF of 2118 bp encoding a deduced protein with 705 amino acids, and the second one 2148 bp coding for a deduced protein of 715 amino acids. Comparative analysis reveals that both isoforms belong to the α-type, and are named SG2NAα and SG2NAα^+^. RT-PCR and western blot analysis reveal that the SG2NA gene is differentially expressed in 9 tissues examined. During goldfish development, while the SG2NA mRNAs remain relatively constant in the first 3 stages and then become decreased and fluctuated from gastrula to larval hatching, the SG2NA proteins are fluctuated, displaying a peak every 3 to 4 stages. Each later peak is higher than the earlier one and the protein expression level becomes maximal at hatching stage. Together, our results reveal that SG2NA may play an important role during goldfish development and also in homeostasis of most adult tissues.

## Introduction

Protein serine/threonine phosphatase 2A (PP-2A) is a major eukaryotic phosphatase, regulating many different physiological functions including metabolic homeostasis, DNA replication, gene transcription, RNA splicing and maturation, protein translation, cell cycle progression, cell senescence and apoptosis, morphogenesis, development, and cell transformation.^1^–^7^

PP-2A exists either in a core enzyme or in a holoenzyme.^5^,^8^ The core enzyme consists of a 65 kDa scaffolding protein known as A subunit tethering a 36 kDa catalytic C subunit.^8^ Both A and C subunits exist in α and β isoforms encoded by different genes.^5^ The full specific activity towards a certain substrate of PP-2A core enzyme is achieved through binding of a variable regulatory subunit to form the heterotrimeric holoenzyme.^5^ Early studies have established that members in 3 subfamilies, B, B′, and B″, can contribute to the regulatory subunit for PP-2A.^4^,^5^ More recently, a new subfamily of proteins including striatin, SG2NA and zinedin have been shown to directly interact with PP-2A.^9^ These proteins bind to PP-2A in the absence of the regulatory subunits from the B, B′, or B″ subfamilies and can act as substrates for PP-2A.^10^,^11^ Thus, it is suggested that the striatin family members may become the fourth subfamily of regulatory subunits for PP-2A.^10^,^11^

While both striatin and zinedin are predominantly expressed in the brain, SG2NA is expressed in many different tissues including heart, skeleton muscle, brain and lung.^13^ Thus, besides the role in directing specific PP-2A activity, SG2NA is suggested to have other functions.^9^ Indeed, yeast two hybrid-selection has revealed that SG2NA can interact with another protein named phocein, which is found functioning in membrane traffic within dentritic spines.^14^ In addition, it is found that SG2NA can act as a molecular scaffold to promote localization of the estrogen receptor (ER)α to the plasma membrane and organize the ER-eNOS membrane signaling complex in endothelial cells.^15^

To explore the possible functions of SG2NA in animal development, we have isolated two SG2NA cDNAs from goldfish, a model organism widely used for in *vivo* studies.^16^–^18^ Our results demonstrate that the goldfish SG2NA cDNAs encode two deduced proteins, which belong to the α-Type isoform and are named SG2NAα and SG2NAα^+^. RT-PCR reveals that the SG2NA mRNAs appear relatively constant from cleavage to larval hatching stages during goldfish development. However, the SG2NA proteins as revealed by western blot analysis, show distinct fluctuations, displaying a peak every 3 to 4 stages. Each later peak is higher than the earlier one and the protein expression level becomes maximal at hatching stage. Such distinct patterns of expression not only suggest possible translational and posttranslational control of the SG2NA gene expression during goldfish development but also indicate their important roles in controlling goldfish development. Our demonstration that SG2NA forms complex with JNK1 also supports its role in regulating goldfish development.

## Results

### Molecular cloning of the two SG2NA cDNAs

**Figure 1:**
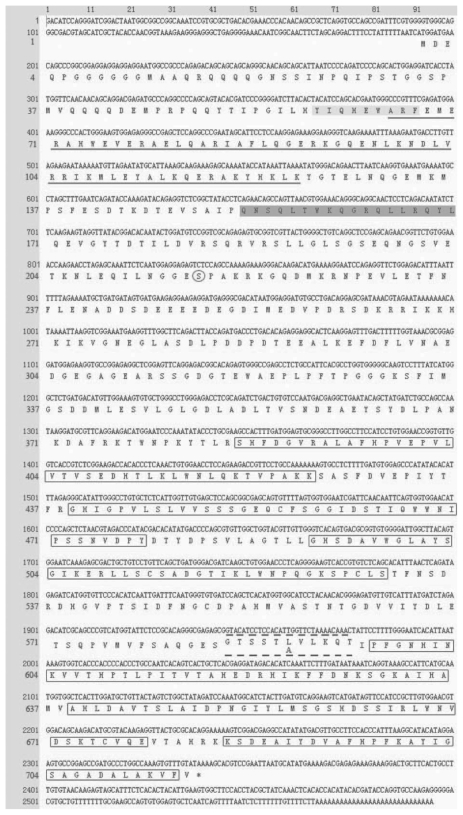
The two full length SG2NA cDNAs and the deduced protein sequences The SG2NAα cDNA sequence is composed of 2,565 bp including a 5′ UTR of 191 bp, a 3′ UTR of 256 bp and a CDS of 2118 bp. The SG2NAα^+^ cDNA contains 2,595 bp including a 5′ UTR of 191 bp, a 3′ UTR of 256 bp and a CDS of 2148 bp (including the nucleotides high-lighted by dash line). The two cDNAs encode two deduced proteins with 705 and 715 amino acids, respectively which are detected in western blot analysis (Figs. 3 to 6). The four conserved domains were high-lighted: the caveolin-binding domain by light grey box, the coiled-coil domain by solid underline, the calmodulin-binding domain by dark-shadowed box, and the six WD-40 repeats in the carbonyl terminus by open box. The conserved phosphorylation residue is circled.

**Figure 2:**
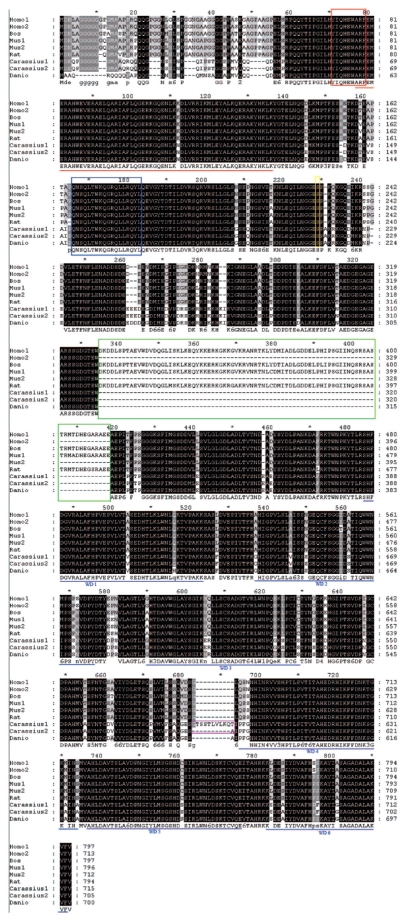
Alignment of the deduced Goldfish SG2NA amino acid sequences with known human, bovine, mouse, rat and zebrafish SG2NA amino acid sequences Homo1, Homo sapiens SG2Nβ-isoform (NM_001083893); Homo2, Homo sapiens SG2Nα-isoform (NM_014574); Bos taurus, bovine SG2NAβ (BC140553); Mus1, mouse SG2NAβ (NM_052973); Mus2, mouse SG2NAα (BC138055); Rattus, rat SG2NAβ (NM_001029897); Carassius 1, goldfish SG2NAα^+^; Carassius 2, goldfish SG2NAα; Danio, zebrafish SG2NAα (BC155853). The four conserved domains were high-lighted: the caveolin-binding domain by red line box, the coiled-coil domain by red underline, the calmodulin-binding domain by blue box, and the six WD-40 repeats in the carbonyl terminus by blue lines. The unique 10 amino acids in SG2NAα^+^ is high-lighted by purple box. The yellow box highlights the conserved phosphorylation serine residue and the green box points out the variation region among different SG2NAhomologues.

**Figure 3:**
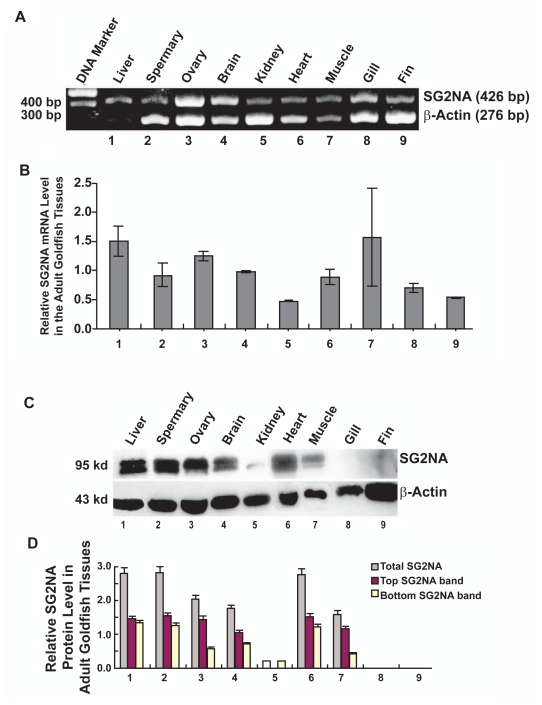
Tissue-specific differential expression of SG2NAα and SG2NAα^+^ mRNAs and proteins in adult goldfish **A**) RT-PCR to detect the total mRNA levels of SG2NAα and SG2NAα^+^ in 9 tissues of adult goldfish as indicated. The primers and the amplification conditions used in the present study are described in Table 1 and 2. Both the SG2NA primers and the β-actin primers were added to the same reaction at the begining of PCR. The specific SG2NA primers amplify the same 426 bp DNA band of both SG2NAα and SG2NAα^+^ cDNAs. As the internal control, a 276 bp β-actin DNA band was also amplified. **B**) Quantitative analysis of the RT-PCR results presented in A and those not shown (averaged from three experiments). The relative level of expression (fold) was calculated by dividing the total pixel from each SG2NA RNA band with the total pixel from the corresponding β-actin mRNA band. **C**) Western blot analysis to detect the protein levels of SG2NAα (bottom band) and SG2NAα^+^ (top band) in 9 tissues of adult goldfish as indicated. Fifty μg of total proteins extracted from 9 tissues of the adult goldfish were separated by 10% SDS-PAGE, transferred to nitrocellulose membranes, and probed with an antibody against SG2NA (top panel) or an antibody recognizing β-actin (bottom panel) at 1:500 dilution at 4 °C overnight. After three washes with TBS-T (10 mM Tris, pH 8.0; 150 mM NaCl, 0.05% Tween 20), the blots were incubated for 45 min with anti-goat IgG (1:1000 dilution), which was linked to peroxidase. At the end of the incubation, the blots were washed twice with TBS-T followed by another two washes with TBS (10 mM Tris, pH 8.0; 150 mM NaCl) and finally visualized with the Amersham ECL kit. Molecular weight was determined according to the protein markers obtained from Fermentas (CAT #0671) **D**) Quantitative results of the two protein isoforms: SG2NAα (yellow bar for bottom band) and SG2NAα^+^ (red bar for top band) from three independent experiments. After exposure, the bands shown in C were processed with the automated digitizing system from the Silver Scientific Corporation. The relative level of expression (fold) was calculated by dividing the total pixel for each SG2NA band with the total pixel from the corresponding β-actin band. The grey bar represents the total SG2NA level (SG2NAα + SG2NAα^+^).

**Figure 4:**
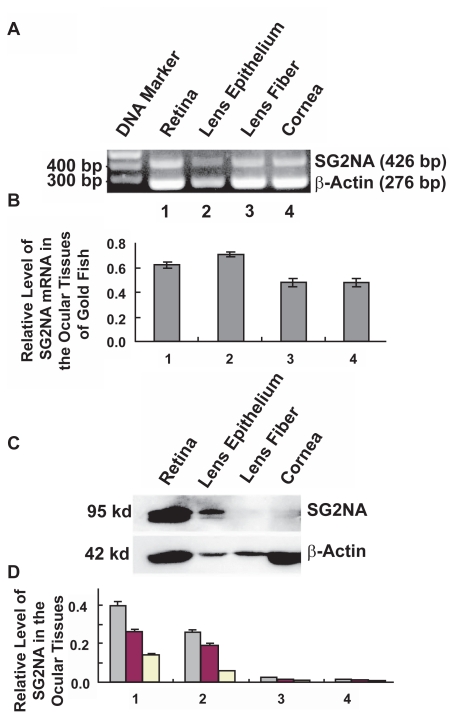
Differential expression of SG2NA in the four major ocular tissues of adult goldfish **A**) RT-PCR to detect the mRNA levels of SG2NAs in four ocular tissues of the adult goldfish as indicated. The primers and the reaction conditions are the same as described in Figure 3. **B**) Quantitative results of the SG2NA mRNA expression. The quantification method used was the same as described in Figure 3. Note that retina and lens epithelium have relatively higher levels of SG2NA mRNAs. **C**) Western blot analysis to detect the SG2NA protein levels in four ocular tissues of the adult goldfish as indicated. **D**) Quantitative results of the SG2NA protein expression from 3 independent experiments. The quantification method was the same as described in Figure 3. Note that the SG2NA proteins are strongly expressed in retina, but much reduced in lens epithelium, and just barely detectable in lens fiber and cornea. Grey bar: total SG2NA; Red bar: SG2NAα^+^; Yellow bar: SG2NAα.

**Figure 5:**
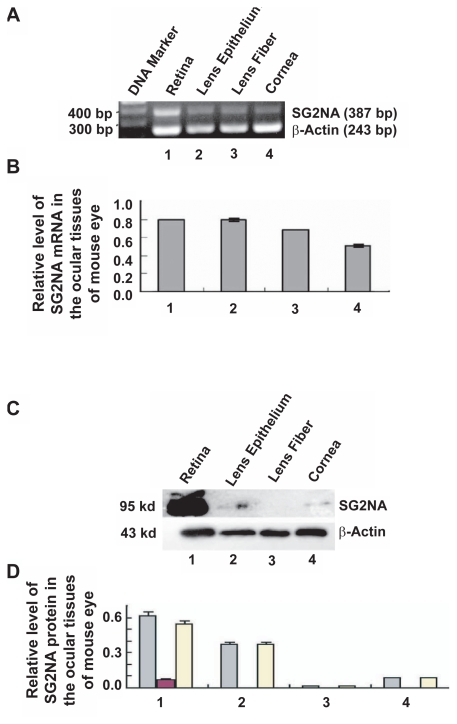
Differential expression of SG2NA in the four major ocular tissues of adult mouse **A**) RT-PCR to detect the mRNA levels of SG2NAs in four ocular tissues of the adult mouse as indicated. The primers and the reaction conditions are described in Table 1 and Table 2. **B**) Quantitative results of the SG2NA mRNA expression from three experiments. The quantification method used was the same as described in Figure 3. Note that both retina and lens epithelium of mouse eye display similar levels of SG2NA mRNAs. **C**) Western blot analysis to detect the SG2NA protein levels in four ocular tissues of the adult mouse as indicated. **D**) Quantitative results of the SG2NA protein expression from three independent experiments. The quantification method was the same as described in Figure 3. Note that the SG2NA proteins are also strongly expressed in retina, but much reduced in lens epithelium and cornea, and just barely detectable in lens fiber. Grey bar: total SG2NA; Red bar: SG2NAα^+^; Yellow bar: SG2NAα.

**Figure 6:**
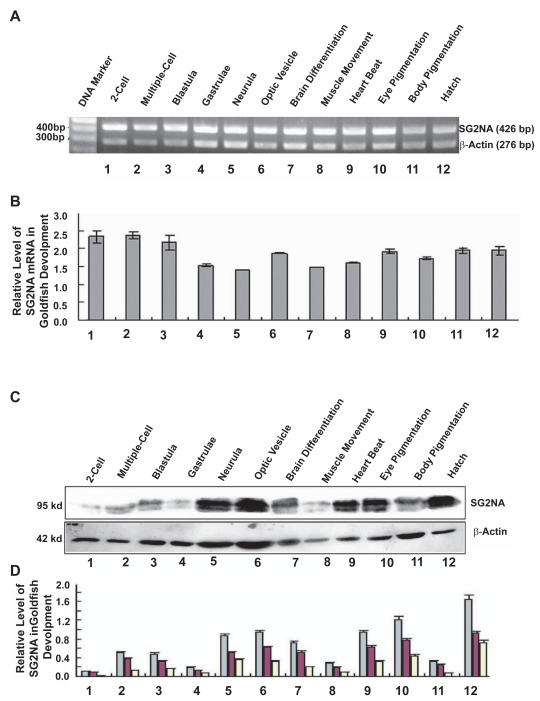
Temporal expression patterns of SG2NA gene during embryonic development of goldfish **A**) RT-PCR to detect the mRNA levels of SG2NA during 12 different developmental stages. The primers used and the reaction conditions are the same as described in Figure 3. **B**) Quantitative results of the SG2NA mRNA expression from three independent experiments. The Quantification method is the same as described in Figure 3. Note that the SG2NA mRNA levels are maintained in relatively constant from two-cell stage to blastula, then decreased to 60% at the gastrula and neurula stages and become fluctuated from optic vesicle to hatching larvae. **C**) Western blot analysis to detect the protein expression levels SG2NAα and SG2NAα^+^ during 12 development stages of goldfish shown in A. The experimental procedures are the same as described in Figure 3. **D**) Quantitative results of the two protein isoforms: SG2NAα (yellow bar for bottom band) and SG2NAα^+^ (red bar for top band) from three independent experiments. The quantification method used was described in Figure 3. Note that the SG2NA proteins are low at 2-cell stage, clearly increased in multiple cell and blastula stages, and then decreased at gastrula stage, this is the first low-high-low cycle. The SG2NA proteins are significantly increased at neurula stage and peaked at optic vesicle stage, then gradually decreased at brain differentiation stage and then at the muscle movement stage, they dropped to the levels of gastrula stage, this is the 2nd cycle. The second peak at optic vesicle stage is much higher than the first peak at multiple cell-blastula stages. After dropping down at muscle contraction, the SG2NA proteins are increased at heart beat stage and peaked at eye pigmentation stage, then drop down again at the body pigmentation stage. This is the 3rd cycle. The peak at the eye pigmentation is higher than that at the optic vesicle stage. From the decreased levels at the body pigmentation, the SG2NA proteins reach maximal expression levels at the hatching larvae. This distinct protein expression pattern is greatly contrast to the relatively steady SG2NA mRNA expression pattern, clearly indicating either differential translation of the SG2NA mRNAs at different stages, or differential stability of the SG2NA proteins at different stages.

Using 3′- and 5′-RACE, we isolated two full length goldfish SG2NA cDNAs, named SG2NAα and SG2NAα^+^ (Fig. 1). The difference between the two cDNAs is the absence or presence of a 30-nucleotide fragment (GTACATCCTCCACATTGGTTCTAAAACAAA) coding for 10 amino acids (GTSSTLVLKQT) located right before the 4th WD repeat. (Fig. 1). The full length SG2NAαcDNA contains 2565 bp with an open reading frame of 2118 nucleotides encode a deduced protein of 705 amino acids. The full length SG2NAα^+^ cDNA consists of 2595 bp with an open reading frame of 2148 nucleotides code for a deduced protein of 715 amino acids. The two proteins encoded by the two cDNAs were confirmed by Western blot analysis (Figs. 3 to 6). The amino acid sequence alignment analysis through ExPASy and other sequence analysis program revealed that both proteins comprise four protein-protein interaction motifs. From N- to C-terminus, the four motifs are the caveolin binding motif, the coiled-coil structure, the calmodulin-binding domain and 6 WD tandem repeats (Fig. 2). In addition, similarity comparison suggests that the conserved Ser-216 residue would undergo phosphorylation modulation (circled amino acid in Fig. 1). Alternative splicing variants from exon 8 and exon 9 of SG2NA mRNAs have been detected in human and mouse,^9^,^10^,^13^ and the two goldfish SG2NA cDNAs reported here belong to α type isoform. The amino acid sequence alignment analysis also shows that the goldfish SG2NA protein (SG2NAα) share high levels of homogeneity with that from zebrafish, human and mouse with amino acid identity of 94.3%, 79.5% and 79.8, respectively (Fig. 2).

### Tissue-specific expression of SG2NA in liver, testis, ovary, brain, kidney, heart, muscle, gill and fin

To explore the possible functions of SG2NA in various tissues of the lower vertebrates, we first examined the mRNA expression of the SG2NA gene in 9 tissues from the goldfish using reverse transcription-linked polymerase chain reaction (RT-PCR) analysis. As shown in Figure 3A, a strong band of 426 bp from goldfish SG2NA specific primers was detected in the ovary and brain. A quantitative analysis of the RT-PCR results from three independent experiments revealed that the goldfish liver and muscle displayed the highest expression levels of SG2NA mRNAs (Fig. 3B). A reduced level of the same band was detected in the ovary, brain, heart and spermary. A further reduced level of the same band was found in the kidney, gills and fins (Fig. 3B). The ubiquitous SG2NA mRNA expression pattern in adult goldfish tissues is consistent with the previous report about the SG2NA expression in mouse.^13^ However, the demonstration of the SG2NA mRNA in genital glands in Figures 3A and 3B is the first report.

To explore the tissue-specific protein expression patterns of the SG2NA gene, we conducted western blot analysis. As shown in Figures 3C and 3D, most tissues expressing SG2NA proteins contain both SG2NAα and SG2NAα^+^ two forms. The kidney only expresses SG2NAα but neither in the gill and fin tissues as revealed from three independent experiments. Among the tissues expressing SG2NA, the highest expression levels of the SG2NA proteins were detected in the liver, spermary and heart (Figs. 3C and 3D). A reduced level of this protein was detected in ovary, brain and skeletal muscle (Figs. 3C and 3D). A much reduced SG2NA protein expression was found in kidney. The obvious difference in the expression levels between mRNAs and proteins of SG2NA in various tissues of goldfish, especially in kidney, gill and fin, indicated presence of differential translation of SG2NA mRNAs or differential stability of the SG2NA proteins (Figs. 3A and 3C).

As shown in Figures 3C and 3D, SG2NAα and SG2NAα^+^ display differential expression in different tissues. In liver, spermary and heart, the two isoforms displayed similar levels of expression. In ovary and skeletal muscle, however, expression of the SG2NAα^+^ isoform was much higher than that of the SG2NAα isoform. As mentioned above, in kidney, we only detected the SG2NAα isoform.

### Differential expression of SG2NA in major ocular tissue of goldfish

Early studies have shown that certain regulatory subunits of PP-2A may have important functions in eye development and pathology.^19^,^20^ To explore the possible functions of SG2NA in vertebrate eye, we examined its expression in 4 major ocular tissues of goldfish: retina, lens epithelium, lens fiber and cornea. As shown in Figures 4A and 4B, RT-PCR revealed that the highest levels of SG2NA mRNAs were found in goldfish lens epithelium and retina. A reduced levels of SG2NA mRNAs were detected in the lens fiber and cornea. For the proteins, the highest levels of SG2NAs were found in retina (Figs. 4C and 4D). The expression levels of SG2NA proteins were much reduced in the lens epithelium (Figs. 4C and 4D). In contrast to both retina and lens epithelium, goldfish lens fibers and cornea contained hardly detectable SG2NA proteins (Figs. 4C and 4D). In both retina and lens epithelium, more SG2NAα^+^ than SG2NAα was found (Fig. 4D).

### Differential expression of SG2NA in major ocular tissue of mouse eye

To further explore the differential expression patterns of SG2NA mRNAs and proteins in vertebrate eye, we examined their expressions in the same four tissues of mouse eye.

As shown in Figures 5A and 5B, an approximately equal levels of SG2NA mRNAs were observed in mouse retina and lens epithelium. A reduced level of SG2NA mRNAs were detected in mouse lens fiber and a further reduced level of SG2NA mRNAs were found in mouse cornea (Figs. 5A and 5B). At the protein level, however, the SG2NA protein expression patterns were very similar to that in goldfish. As shown in Figures 5C and 5D, the highest expression levels of SG2NA proteins were found in mouse retina. A reduced level of SG2NA protein (SG2NAα) was detected in mouse lens epithelium (Figs. 5C and 5D). A much reduced level of SG2NA protein (SG2NAα^+^) was observed in mouse cornea but no detectable SG2NA protein was found in lens fibers (Figs. 5C and 5D).

### Expression of SG2NA during goldfish development

Although some orthologue members of the striatin family have been shown to play important role in development of invertebrates,^21^,^22^ it remains to be elucidated if SG2NA plays a role during vertebrate development. For this reason, we investigated the expression patterns of SG2NA at twelve different developmental stages: the two-cell, multiple-cell, blastula, gastrula, neurula, optic vesicle, brain vesicle, muscle movement, heart beat, eye pigmentation, body pigmentation and hatching larval stages. As shown in Figures 6A and 6B, SG2NA mRNA was maintained at relatively high level from two-cell to blastula, indicating the maternal source of the SG2NA mRNAs. Then, the SG2NA mRNA level was reduced to about 60% at the gastrula stage; and further reduced to approximately 56% at the neurula stage when compared with that at the first three embryonic stages. This mRNA level was transiently increased at the optical vesicle stage, and then dropped down slightly at the brain differentiation stage and then became slightly increased at the body pigmentation stage and further increased at the muscle movement stage and thereafter it was maintained at the similar level from heart beat to hatch stages, a status reflecting new mRNA synthesis.

In contrast to the relatively steady levels of SG2NA mRNAs at different stages of goldfish embryos, the SG2NA protein levels varied from one stage to another during goldfish development. As shown in Figures 6C and 6D, western blot analysis revealed that only a trace of SG2NAα^+^ protein was detected at 2-cell stage. As development proceeded, the SG2NA proteins were gradually up-regulated from multiple-cell embryo to blastula, but were briefly decreased at gastrula stage. Then, the SG2NA proteins were up-regulated to the new level at the neurula stage and this level was maintained through optic vesicle and brain differentiation stages. At the muscle contraction stage, the SG2NA proteins became downregulated again and were then further up-regulated from the heart beating to eye pigmentation stage. After a brief dropping down at the body pigmentation stage, the SG2NA proteins were further up-regulated to the maximal level.

### SG2NA Interacts with JNK

**Figure 7:**
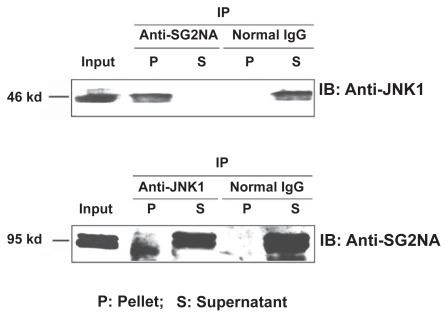
Demonstration that SG2NAs interact with JNK1 Total protein were extracted from goldfish embryos at neurula stage and then used for immunoprecipitation using anti-SG2NA antibody, or anti-JNK1 antibody as indicated. Normal IgG was used as control. The immunoprecipitated proteins were used for western blot analysis using anti-JNK1/2 antibody or antibody against SG2NA as indicated.

Previous studies have shown that the *Drosophila* CKA protein, an orthologue member of the striatin family can act as scaffold to mediate JNK signaling pathway during embryonic development.^21^ To explore if SG2NA has similar functions, we conducted co-immunoprecipitation with total proteins extracted from goldfish embryos of neurula stage. As shown in Figure 7, SG2NA and JNK1 could be co-immunoprecipitated, indicating that they can form *in vivo* interacting complex. Thus, it is possible that SG2NA may not only modulate PP-2A functions but also regulating JNK signaling pathway.

## Discussion

In the present study, we have shown that 1) the goldfish SG2NA gene generates two cDNAs with ORFs of 2118 bp and 2148 bp, coding for the deduced proteins of 705 and 715 amino acids, respectively; 2) the deduced goldfish SG2NA proteins share an amino acid homologue of 94.3%, 79.5% and 79.8% to that from zebrafish, human and mouse, respectively; 3) the SG2NA mRNAs are present in all nine tissues examined but the SG2NA proteins are only expressed in 7 tissues; 4) In the ocular tissues, the SG2NA proteins are highly expressed in retina, intermediately in lens epithelium and much lower in cornea and lens fiber cells; 5) During Goldfish development, while the mRNAs are maintained at a relatively steady level at different stages, the SG2NA proteins fluctuate 3 times. Together, our results reveal that expression of the SG2NA gene displays distinct tissue-specificity and also unique developmental patterns which indicate that they may have important functions controlling animal development and maintaining adult tissue homeostasis of goldfish.

### Structural features of goldfish SG2NA

**Figure 8:**
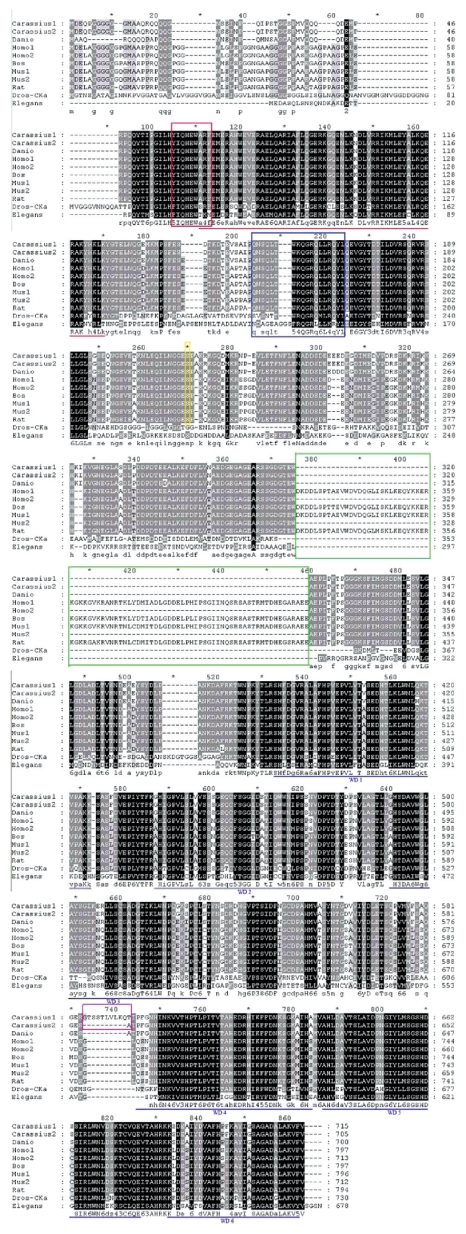
Alignment of the deduced Goldfish SG2NA amino acid sequences with vertebrates SG2NA and invertebrate SG2NA orthologue sequences Carassius 1, goldfish SG2NAα^+^; Carassius 2, goldfish SG2NAα; Danio, zebrafish SG2NAα (BC155853); Homo1, Homo sapiens SG2Nβ-isoform (NM_001083893); Homo2, Homo sapiens SG2Nα-isoform (NM_014574); Bos, bovine SG2NAβ BC140553); Mus 1, mouse SG2NAβ (NM_052973); Mus2, mouse SG2NAα (BC138055); Rat, rat SG2NAβ (NM_001029897); Dros-Cka, *Drosophila* Cka (NM_164781); Elegans, *C. elegans* SG2NA (NM_073263). The four conserved domains were high-lighted: the caveolin-binding domain by red line box, the coiled-coil domain by red underline, the calmodulin-binding domain by blue box, and the six WD-40 repeats in the carbonyl terminus by blue lines. The yellow box highlights the conserved phosphorylation serine residue and the green box points out the variation region among different SG2NA homologues. The unique 10 amino acids in SG2NAα^+^ is high-lighted by purple box.

The SG2NA belongs to the striatin family,^9^ which consists of Striatin, SG2NA, Zinedin and *Drosophila* CKA four members. The gene coding for SG2NA has been cloned from human (NM_001083 893 and NM_014574), bovine (BC140553), mouse (NM_052973 and BC 138055), rat (NM_001029897), and zebrafish (BC155853). Two major isoforms, SG2NAα and SG2NAβ have been identified for SG2NA in human and mouse.^13^ The SG2NAα. short form containing 713 amino acids in human and 712 amino acids in mouse; and SG2NAβ is the longer form containing 797 amino acids in human and 796 amino acids in mouse.^13^ The two isoforms are derived from alternative splicing with the SG2NAβ form containing exons 8 and 9 but missed in the SG2NAα form.^13^ Compared with SG2NAα and SG2NAβ two isoforms in human and mouse, the two isoforms we isolated from goldfish contain 705 and 715 amino acids, both close to the SG2NAα. Thus, we named the 705-amino acid SG2NA as SG2NAα, and the 715-amino acid SG2NA as SG2NAα^+^. The SG2NAα^+^ has a 10 amino acid insertion at a position just before the 4th WD-repeat, which is not found in other species (Fig. 8). Both forms are expressed in most tissues including liver, heart, brain, muscle, spermary and ovary though kidney only expresses SG2NAα (Fig. 3C). During goldfish development, both SG2NAα and SG2NAα^+^ are present in all stages except for the two-cell stage (Fig. 6C), indicating both isoforms are functional. Since SG2NAα and SG2NAα^+^ lack exons 8 and 9, we predict that they come from a different alternative splicing beyond exon 9. Alternative splicing of SG2NA in other exon has been recently identified in mouse where five novel splice variants have been reported. Of the five variants, two are devoid of the carboxyl terminal WD-40 repeats. These variants of SG2NA are generated by alternative splicing at exon 7–9 regions and differ in their expression profiles in various tissues tested.^23^ In goldfish, where the alternative splicing may occur remains to be determined.

The SG2NA protein contains 4 major domains. From N-terminal to C-terminal, they are caveolin binding domain, coiled-coil structure, calmodulin binding domain, and a large WD-repeat domain.^9^ The caveolin binding domain and the calmodulin binding domain are completely conserved among goldfish and other vertebrates (human, bovine, mouse, rat and zebrafish), but less than 80% conservations are found when compared with the orthologues in *Drosophila* and *C. elegans* (the red and blue boxes in Figs. 2 and 8). The coiled-coil structure is completely conserved between goldfish and zebrafish (high-lighted by red line in Figs. 2 and 8). However, there is one amino acid difference between fish and other higher vertebrates (human, mouse, rat and bovine; marked by red line in Figs. 2 and 8). Distinct difference exists in this domain among goldfish SG2NAs, *Drosophila* CKA and *C. elegans* SG2NA (high-lighted sequence by red line in Fig. 8). The SG2NA proteins from different vertebrates contain six WD repeat domains in the C-terminal. In WD repeat 1, one amino acid difference is found between goldfish and zebrafish, and also between goldfish and other higher vertebrates (human, mouse, rat and bovine; high-lighted by blue line). In all other WD repeats, more than one amino acid difference has been detected (blue line high-lighted segments in Figs. 2 and 8). The conservation of these structural domains or motif between goldfish and other vertebrates suggest that the basic functions of SG2NA are well conserved. According Moreno et al^10^,^11^ striatin and SG2NA may target the catalytic subunit C of PP-2A to components of Ca^2+^-dependent signaling pathways. This interesting suggestion is based upon the fact that striatin, SG2NA, and PP2A subunit C were found to co-immunoprecipitate within detergent complexes from lysed NIH-3T3 cells. Indeed, in goldfish, SG2NA and the catalytic subunit C of PP-2A can be co-immunoprecipitated (data not shown). On the other hand, the catalytic subunit of PP-2A undergoes two types of post-translational modification: phosphorylation and methylation, which are important regulatory processes for the binding of the B type subunits.^24^ Methylation of the carboxyl-terminus of the C subunit is critical for the association with B type subunits.^24^ Although striatin and SG2NA directly interact with PP-2A holoenzyme in the absence of the B, B′ or B″ regulatory subunits,^10^ they bind to both methylated and unmethylated C subunits and mutations in the binding domain for B on the C subunit do not prevent striatin and SG2NA binding.^24^ When PP-2A is inhibited by okadaic acid, phosphorylation of striatin and SG2NA becomes dramatically increased, suggesting the two proteins are substrates of PP-2A holoenzyme.^11^ Together, these results suggest that striatin and SG2NA may modulate PP-2A activity by sequestering PP-2A holoenzyme in specific subcellular compartments or specific signaling pathways. Besides its role through the association with PP-2A, SG2NA may also provide scaffold for other signal complex. In the present study, we demonstrated that SG2NA can be co-immunopreciptated with JNK1 kinase (Fig. 7). This result is consistent with early studies where it has been shown that the *Drosophila* orthologue member, CKA of the striatin family which shares 56% homology with striatin or SG2NA at the protein level, forms a physical complex with several kinases including HEP and BSK, and also the components of AP-1 family including JUN and FOS.^21^,^25^

### SG2NA may play an important role in goldfish development

In the present study, we have shown that the two mRNAs for SG2NAα and SG2NAα^+^ are maintained at relatively steady levels (Figs. 6A and 6B). In contrasting, expression of the SG2NA proteins appeared in several waves with each wave covering 3 to 4 different stages (Figs. 6C and 6D). The relative constant in the goldfish SG2NA mRNA levels but the distinct fluctuations in the protein levels suggest the presence of either differential translation of the SG2NA mRNAs, or differential stability of SG2NA proteins, or both. More importantly, the presence of different waves of SG2NA protein expression indicates that the SG2NA proteins are strictly regulated at each developmental stage and presumably their functions can be finely tuned up through their differential expression.

Although the exact functions of SG2NA in development remain to be elucidated, the crucial role of striatin in development has been explored in cultured cells from rat. Bartoli and collaborators^26^,^27^ have shown that a reduction of around 80% of the striatin by anti-sense oligonucleotides in cultured spinal cord motoneurons dramatically impairs rat dendritic outgrowth. In *Drosophila,* as mentioned above, Chen et al^21^ have shown that the orthologue member of the striatin family, CKA, is an important scaffold protein, organizing a molecular complex of kinases and transcription factors. The absence of CKA induces a dorsal-open cuticle phenotype and cell death. The embryos display head defects and lack the dorsal epidermis. Such a phenotype is totally rescued by *C. elegans* or mammalian orthologues of CKA.^21^ Another non-vertebrate orthologue of the striatin family is the PRO11 protein isolated from filamentous fungi, *Sordaria macrospora.*^22^ This protein has 17% homology with mammalian members and27% homology with *Drosophila* CKA. Mutation of *pro11* causes sterility, which can be rescued by mouse striatin complementation.^22^ Whether the SG2NAs in goldfish have similar functions in regulating animal development is currently under investigation.

### SG2NA may have important functions in vertebrate eye

In the present study, we have demonstrated high levels of SG2NA proteins are present in retina from both goldfish and mouse. Such a strong expression suggests that SG2NA may play an important role in this ocular tissue. How might SG2NAs exert functions in retina? One possibility is that the SG2NA may regulate the visual cycle in photoreceptor. It has been shown that the opsin-mediated phototransduction in photoreceptors requires the function of PP-2A,^28^ SG2NA may act as a regulatory subunit of PP-2A in retina to modulate the phototransduction cycle.

SG2NAs may be also involved in regulation of other signaling pathways in the retina. For example, Rorick et al^19^ have shown that one of the B family subunits for PP-2A is required for the IGF/PI3K/Akt pathway and that interfering with the PI3K/Akt pathway inhibits eye induction. Moreover, during eye field separation, this subunit is also implicated in regulating the hedgehog-signaling pathway. Whether goldfish SG2NAs could regulate these signaling pathways remains to be determined.

In summary, the goldfish SG2NA gene encodes two cDNAs which may be derived from the alternative splicing in a new exon. Our demonstration that SG2NA fluctuates during goldfish development indicates its important role in regulating the developmental process. The strong expression of SG2NAs in retina also suggests their critical role in this ocular tissue. The presence of the interaction between SG2NA and JNK1 suggests that SG2NA not only modulates functions of PP-2A and also other signaling complexes.

## Experimental Procedures

### Chemicals

The RNA extraction kit was purchased from Omega. The reverse transcription kit was obtained from Invitrogen, Inc. The protein size marker was obtained from Fermentas. The 5′ and 3′ RACE cloning kit was purchased from Clontech, Inc. PCR Taq polymerase and the PMD18-T vector were obtained from Takara Inc. Anti-SG2NA antibody and anti-goat IgG antibody were purchased from Santa Cruz Biotechnology. Anti-beta-actin antibody was obtained from Sigma, Inc. Gel purification kit and all the oligo primers were obtained from Sangon, Inc.

### Animals

The goldfish of 6 months to one year in age were collected from the Experimental Fish Culture Facility of the Key Laboratory of the Educational Ministry of China in Hunan Normal University and fertilization was conducted in the laboratory. Mice of 4 weeks were obtained from Hunan Normal University animal facility.

### Collection of tissues and embryos

Goldfish were sacrificed through removal of gill tissues. Various tissues including liver, spermary, ovary, brain, kidney, heart, muscle, gill and fin were quickly dissected out on ice and then frozen under liquid nitrogen for homogenization first with a mortar and then with 1 ml syringe (18.5G and 23.5 needles passed).

Artificial fertilization was conducted in Hoff’s solution (0.1 g CaCl_2_, 0.05 g KCl, 3.5 g NaCl dissolved in 1000 ml distilled H_2_O). The fertilized egg membranes were removed with 0.4% pancreatic protease and the de-membraned eggs were allowed to develop at 22 °C in Hoff’s solution. Under microscopic examination, the developing embryos at stages of 2-cell, multiple-cell, blastula, gastrula, neurula, optical vesicle, brain differentiation, muscle movement, heart beat, eye pigmentation, body pigmentation and hatch were collected and frozen under liquid nitrogen. The frozen embryos were homogenized for extraction of RNA and proteins as described below.

### Preparation of total RNAs from various tissues of mouse eye

For collection of mouse eye tissues, mice were euthanatized by CO_2_ inhalation. The eyeballs were removed and various components of eye tissues were carefully dissected by a posterior approach.^29^,^30^ The retina, lens capsule/epithelial cells, lens fiber cells and cornea were removed immediately and transferred into Eppendorf tubes containing 500 μl RNA extraction buffer (Trizol, Gibco BRL CAT# 15596–026) and were homogenized on ice with an Eppendorf tube micropestle (Brinkmann Instruments, Inc.). The remaining procedures of RNA extraction were the same as previously described.^29^,^30^

### Reverse transcription-linked polymerase chain reaction (RT-PCR)

Reverse transcription was conducted using a kit from Invitrogen (Invitrogen #18085–019) as previously described.^29^ Briefly, 2 μg of total RNA were used in a total reaction volume of 20 μl. For PCR amplification, all the primers used were listed in Table 1. Two μl of the reverse transcription reaction mixture were used for PCR reaction. For detection of SG2NA expression, Gexp primer pairs (for goldfish) and Mexp primer pairs (for mouse) were used. And goldfish β-actin and mouse β-actin primers were used as control for goldfish and mouse SG2NAs, respectively. Both Gexp primers and goldfish β-Actin primers, or Mexp primers and mouse β-Actin primers were added at the begining of PCR into the same reaction tube and the PCR reaction was continued 30 cycles. Each cycle was run with the program listed in Table 2. At the end of each reaction, the PCR products were separated by agarose gel (1.5%) electrophoresis and photographed under UV illumination.

## Molecular Cloning of the Full Length SG2NA cDNA

RT-PCR cloning strategy used to clone the full length SG2NA cDNA from goldfish was described before.^31^ Two ‘rad’ oligo primers (see Table 1 for DNA sequences) were designed to amplify a conserved fragment of 300 bp goldfish cDNA near the 3′ end of the SG2NA coding region using zebrafish SG2NA gene (XM_686488) as reference. Reverse transcription was conducted with a kit from Invitrogen with 2 μg total RNA from goldfish. Two μl of the RT reaction mixture was used for PCR reaction containing 1 μl of 10 μM rad primers, 10 μl 2X PCR mixture and 7 μl H2O. PCR reaction was run 30 cycles in the following conditions: 94 °C, 5 min; 94 °C, 30 seconds; 58 °C, 30 seconds; 72 °C, 60 seconds for 30 cycles. The amplified products were gel-purified and cloned into PMD18-T vector (TAKARA) for sequencing.

3′ Race was performed using UPM mix solution (longer primer): 0.4 μM 5′-CTAATACGACTCACTATAGGGCAAGCAGTGGTATCAACGCAGAGT-3′, shorter primer: 2 μM 5′-CTAATACGACTCACTATAGGGC-3′) and 3gsop primer (see DNA sequence in Table 1). PCR was performed in a 20 μl reaction mix containing 1 μl of the 3′ cDNA library (Smart Race cDNA reverse transcriptase kit was purchased from Clontech Inc.), 1 μl of mixed UPM short primer and the synthesized 3gsop primer, and 10 μl 2X PCR mix solution. Amplification conditions were: 94 °C 5 min; 94 °C 30 sec, 72 °C 2 min for 5 cycles; 94 °C 30 sec, 65 °C 30 sec, 72 °C 2 min for 30 cycles. The PCR products were re-amplified using 1 μl of 10 μM primer 3gsip (see DNA sequence in Table 1) and NUP (5′AAGC AGTGGTATCAACGCAGAGT-3′). The amplified products were gel-purified and cloned into PMD18-T vector (TAKARA) for sequencing. A DNA fragment of 1600 bp corresponding to 3′ end partial cDNA of SG2NA was obtained.

5′-RACE was performed using the 5-RACE kit. PCR procedures were the same as described using 1 μl of mixed UPM long primer and the synthesized 5gsop primer (see DNA sequence in Table 1) and UPM mix solution. Re-amplification of the PCR products were conducted using 1 μl of 10 μM primer 5gsip (see DNA sequence in Table 1) and UPM long primer. A DNA fragment of 950 bp corresponding to 5′ end partial cDNA of SG2NA was obtained.

### Preparation of total proteins from various ocular tissues of mouse eye

After dissection of various components of mouse eye, they were transferred into an Eppendorf tube containing 200 μl extraction buffer (50 mM Tris-HCl, pH 7.0; 0.1% β-mercaptoethanol; 0.1 mM EDTA, 0.1 mM EGTA, 2 mM leupeptin, 1 mM PMSF, 1 mM benzamidine-HCl, 2 mM DTT, 0.5% Triton X-100) and were homogenized on ice with an Eppendorf tube micropestle (Brinkmann Instruments, Inc.). The remaining procedures were the same as previously described.^29^

### Western blot analysis

Western blot analysis was conducted as previously described.^32^,^33^ Briefly, 50 to 100 μg of total proteins from various ocular tissues of mouse eye were separated by 10% SDS-polyacrylamide gel electrophoresis and transferred into supported nitrocellulose membranes (Gibco BRL). The protein blots were blocked with 5% milk in TBS (10 mM Tris, pH 8.0; 150 mM NaCl) overnight at 4 °C. Then, each blot was incubated with an anti-SG2NA antibody (from Santa Cruz Biotechnology) at a dilution of 1:500 in 5% milk prepared in TBS for 60 minutes at 4 °C with mild shaking. After 3 washes with TBS-T (TBS with 0.05% Tween-20), 15 minutes each, each blot was incubated with a secondary antibody (anti-goat IgG from Santa Cruz Biotechnology) at a dilution of 1 to 1000 for 45 minutes. After two washes with TBS-T followed by another two washes with TBS (15 minutes each), the SG2NA protein was detected with an enhanced chemiluminescence detection kit according to the instruction manual from Amersham.

As reference, after stripping the previous antibody, the blot was re-hybridized with the anti-β-actin primary antibody (1:2000 from Sigma, Inc.). After washing with TBST 3 times, the blot was incubated with the anti-mouse IgG (secondary antibody from GE Health Care, Inc. diluted in 1:1000). After washing with TBST 2 times and TBS one time, the β-actin level was detected as described above.

### Immunoprecipitation

Immunoprecipitation was conducted as described before.^33^ Total proteins extracted from goldfish embryos were used for immunopreciptation. The anti-SG2NA or anti-JNK antibodies (Santa Cruz Technology) were used to precipitate JNKs or SG2NA, respectively. The mixtures were incubated at 4 °C for 1 h with gentle shaking, and then 50 μl of protein A/G plus agarose (Santa Cruz Biotechnology) was added into the initial reaction mixture and incubated overnight under the same condition. After the incubation, the mixture was centrifuged for 15 min at 12,000 rpm at 4 °C. The supernatant for each sample was saved, and the pellet was washed three times with RIPA. Following the final wash, the pellet was processed for western blot described above. For negative control, the normal IgG was used as primary antibody for the immunoprecipitation. Total protein imput was also included for Western blot analysis.

### Quantitation of western blot results

After exposure, the x-ray films were analyzed with the Automated Digitizing System from the Silk Scientific Corporation as previously described.^32^ The relative expression levels (fold) were calculated by dividing the total pixel from each band under investigation by the total pixel from the corresponding β-actin band. The quantitative data were averaged from three independent experiments.

## Figures and Tables

**Table 1:** Oligo primers used for GS2NA cloning and RT-PCR analysis.

	Forward primer		Reverse primer
Goldfish b-β-actin	5′-CCGTGACCTGACTGACTACCTC-3′		5′-ATACCGCAAGATTCCATACCC-3′
Rad	5′-GGCTCAGAGCAGAATGGTTC-3′		5′-TTGAGTGCGTCCTCTGTGTC-3′
3gsip	5′-AATCAAGGTCGGAAATGAAGGTTTG-3′	Nup	5′-AAGCAGTGGTATCAACGCAGAGT 3′
3gsop	5′-GGAGGATGTGCCTGACAGGAGCGATAA-3′	Upm short	5′-CTAATACGACTCACTATAGGGC-3′
5gsip	5′-ATATCGCCCTCATCATCTTCCTCTT-3′	Upm long	5′-CTAATACGACTCACTATAGGGCAAGCAGTGGTATCAACGCAGAGT-3′
5gsop	5′-TCGCTCCTGTCAGGCACATCCTCCA-3′		
Gexp	5′-CTGCTCACGAGGATAGACA-3′		5′-AGTTTGATAGCGTAGGTGG-3′
Mouse β-actin	5′-CACTGCCGCATCCTCTTCCT-3′		5′-ATGCCTGGGTACATGGTGGT-3′
Mexp	5′-AAGAGGGTCAACAGGACAAAC-3′		5′-CACAGGCTCTACAGGATGAAA-3′

* Oligo Nup, Upm long and Upm short were provided by Clonetech SMART RACE kit. Other oligos in the table were designed and synthesized.

**Table 2:** PCR temperature and cycle conditions.

Oligos	Temperature and cycle conditions
Rad	94 °C 5 min; 94 °C 30″, 58 °C 30″, 72 °C 1 min, 30 cycles; 72 °C 10 min, 4 °C hold.
3gsop	94 °C 5 min; 72 °C 2 min, 5 cycles; 94 °C 30″, 65 °C 30″, 72 °C 2 min, 30 cycles; 72 °C 10 min, 4 °C hold.
3gsip	94 °C 5 min; 94 °C 30″, 58 °C 30″, 72 °C 1 min, 30 cycles; 72 °C 10 min, 4 °C hold.
5gsop	94 °C 5 min; 72 °C 2 min, 5 cycles; 94 °C 30″, 63 °C 30″, 72 °C 2 min, 30 cycles; 72 °C 10 min, 4 °C hold.
5gsip	94 °C 5 min; 94 °C 30″, 55 °C 30″, 72 °C 1 min, 30 cycles; 72 °C 10 min, 4 °C hold.
Gexp	94 °C 5 min; 94 °C 30″, 56 °C 30″, 72 °C 1 min, 30 cycles; 72 °C 10 min, 4 °C hold.
Mexp	94 °C 5 min; 94 °C 30″, 58 °C 30″, 72 °C 1 min, 30 cycles; 72 °C 10 min, 4 °C hold.

## References

[1] Olsen JV Global, *in vivo*, and site-specific phosphorylation dynamics in signaling networks Cell 2006 127 635 48 17081983 10.1016/j.cell.2006.09.026

[2] Hunter T Protein kinases and phosphatases: the yin and yang of protein phosphorylation and signaling Cell 1995 80 225 36 7834742 10.1016/0092-8674(95)90405-0

[3] Cohen P The structure and regulation of protein phosphatases Annu Rev Biochem 1989 58 453 508 2549856 10.1146/annurev.bi.58.070189.002321

[4] Mumby MC Walter G Protein serine/threonine phosphatases: structure, regulation, and functions in cell growth Physiol Rev 1993 73 673 99 8415923 10.1152/physrev.1993.73.4.673

[5] Moorhead GBG Trinkle-Mulcahy L Ulke-Lemée A Emerging roles of nuclear protein phosphatases Nature Rev Mol Cell Biol 2007 8 234 44 17318227 10.1038/nrm2126

[6] Yan Q Mao YW Li DWC Binder Hirokawa Windhorst Protein serine/threonine phosphatases in the nervous system Encyclopedia of Neuroscience Springer Heidelberg 2009 4 3325 9

[7] Qin JC Protein phosphatase-2A is a target of epigallocatechin-3-gallate and modulates the p53-Bak apoptotic pathway Cancer Res 2008 68 4150 62 18519674 10.1158/0008-5472.CAN-08-0839

[8] Xu Y Structure of the protein phosphatase 2A holoenzyme Cell 2006 127 1239 51 17174897 10.1016/j.cell.2006.11.033

[9] Benoist M Gaillard S Castets F The striatin family: A new signaling platform in dendritic spines J Physiol 2006 99 146 153 10.1016/j.jphysparis.2005.12.006 16460920

[10] Moreno CS WD40 repeat proteins striatin and S/G(2) nuclear autoantigen are members of a novel family of calmodulin-binding proteins that associate with protein phosphatase 2A J Biol Chem 2000 275 5257 63 10681496 10.1074/jbc.275.8.5257 PMC3505218

[11] Moreno CS A mammalian homolog of yeast MOB1 is both a member and a putative substrate of striatin family-protein phosphatase 2A complexes J Biol Chem 2001 276 24253 60 11319234 10.1074/jbc.M102398200 PMC3503316

[12] Berndt N Roles and regulation of serine/threonine-specific protein phosphatases in the cell cycle Prog Cell Cycle Res 2003 5 497 510 14593745

[13] Castets F Zinedin, SG2NA, and striatin are calmodulinbinding, WD repeat proteins principally expressed in the brain J Biol Chem 2000 275 19970 7 10748158 10.1074/jbc.M909782199

[14] Baillat G Molecular cloning and characterization of phocein, a protein found from the Golgi complex to dendritic spines Mol Biol Cell 2001 12 663 73 11251078 10.1091/mbc.12.3.663 PMC30971

[15] Lu Q Striatin assembles a membrane signaling complex necessary for rapid, nongenomic activation of endothelial NO synthase by estrogen receptor alpha Proc Natl Acad Sci U S A 2004 101 17126 31 15569929 10.1073/pnas.0407492101 PMC534607

[16] Huang L Proteome comparative analysis of gynogenetic haploid and diploid embryos of goldfish (Carassius auratus) Proteomics 2004 4 235 43 14730685 10.1002/pmic.200300553

[17] Zheng CB cDNA cloning and mRNA expression of the genes encoding regulatory subunits α and γ of the B″ family for PP-2A Progress in Natural Sciences 2009 19 36 42

[18] Fu H Molecular cloning and differential expression of the gene encoding the B′δ regulatory subunit of PP-2A SP from goldfish (Carassius auratus) Sci Sin C Life Sci 2009 39 460 8 10.1007/s11427-009-0094-4 19727590

[19] Rorick AM PP2A:B56epsilon is required for eye induction and eye field separation Dev Biol 2007 302 477 93 17074314 10.1016/j.ydbio.2006.10.011

[20] Kantorow M Differential display detects altered gene expression between cataractous and normal human lenses Invest Ophthalmol Vis Sci 1998 39 2344 54 9804143

[21] Chen HW CKA, a novel multidomain protein, regulates the JUN N-terminal kinase signal transduction pathway in Drosophila Mol Cell Biol 2002 22 1792 803 11865058 10.1128/MCB.22.6.1792-1803.2002 PMC135602

[22] Poggeler S Kuck U A WD40 repeat protein regulates fungal cell differentiation and can be replaced functionally by the mammalian homologue striatin Eukaryot Cell 2004 3 232 40 14871953 10.1128/EC.3.1.232-240.2004 PMC329509

[23] Sanghamitra M WD-40 repeat protein SG2NA has multiple splice variants with tissue restricted and growth responsive properties Gene 2008 420 48 56 18571342 10.1016/j.gene.2008.04.016

[24] Yu XX Methylation of the protein phosphatase 2A catalytic subunit is essential for association of Balpha regulatory subunit but not SG2NA, striatin, or polyomavirus middle tumor antigen Mol Biol Cell 2001 12 185 99 11160832 10.1091/mbc.12.1.185 PMC30577

[25] Martin P Wood W Epithelial fusions in the embryo Curr Opin Cell Biol 2002 14 569 74 12231351 10.1016/s0955-0674(02)00369-1

[26] Bartoli M Monneron A Ladant D Interaction of calmodulin with striatin, a WD-repeat protein present in neuronal dendritic spines J Biol Chem 1998 273 22248 53 9712839 10.1074/jbc.273.35.22248

[27] Bartoli M Down-regulation of striatin, a neuronal calmodulin-binding protein, impairs rat locomotor activity J Neurobiol 1999 40 234 43 10413453

[28] Palczewski K The Catalytic Subunit of Phosphatase 2A DephosphorylatesPhosphoopsin Biochemistry 1989 28 415 9 2540796 10.1021/bi00428a001

[29] Liu WB Differential expression of the catalytic subunits for PP-1 and PP-2A and the regulatory subunits for PP-2Ain mouse eye Mol Vis 2008 14 762 73 18432318 PMC2324119

[30] Li DWC Analysis of expression patterns of protein phosphatase-1 and phosphatase-2A in rat and bovine lenses Invest Ophthalmol Vis Sci 2001 42 2603 9 11581206

[31] Chen L PhD Thesis Human Normal University 2009 chapter 4 52 64

[32] Li DWC Protein expression patterns of the signaling molecules for MAPK pathways in human, bovine and rat lenses Invest Ophthalmol Vis Sci 2003 44 5277 86 14638727 10.1167/iovs.03-0348

[33] Yan Q Protein Phosphatase-1 dephosphorylates Pax-6, a transcription factor controlling brain and eye development J Biol Chem 2007 282 13954 65 17374606 10.1074/jbc.M611476200

